# Development of an Equity Metric for Infrastructure Service Performance Assessment

**DOI:** 10.1111/risa.70126

**Published:** 2025-10-09

**Authors:** Abigail L. Beck, Eun Jeong Cha, Walter Gillis Peacock

**Affiliations:** ^1^ Department of Civil and Environmental Engineering University of Illinois Urbana‐Champaign Urbana Illinois USA; ^2^ Department of Landscape Architecture and Urban Planning Hazard Reduction and Recovery Center Texas A&M University College Station Texas USA

**Keywords:** decision‐making, equity, infrastructure, performance assessment, Theil's *T*

## Abstract

Infrastructure service outages are often disproportionately clustered among vulnerable population groups (e.g., low income) who are also often more adversely impacted by these outages due to limited resources. These inequities need to be reduced for community resilience's improvement. Equity is increasingly considered an integral part of risk‐based decision‐making. There is no consensus on equity's definition within the infrastructure context, but equity is best characterized by five dimensions: recognitional, distributional, restorative, procedural, and transgenerational. Few metrics currently accommodate equity robustly, particularly the restorative dimension. Theil's *T*‐derived infrastructure metrics are presented to assess infrastructure service performance inequities for both the restorative and distributional equity paradigms, which can ultimately support infrastructure intervention decisions (e.g., retrofit, system operation) to robustly consider equity. The restorative paradigm necessitates the assessment of differences between distinct groups, whereas the distributional paradigm necessitates an equality assessment across the entire population. The metrics are derived for two infrastructure service performance quality measures represented through robustness and vulnerability. The proposed metrics are implemented in a limited hypothetical electric distribution network to evaluate the metrics’ capability to detect degrees of inequity introduced into the system through varying nodal vulnerability scenarios. The results confirm the metrics’ ability to benchmark inequity and represent an approach for equity's quantification alongside other factors in risk‐based decision‐making, which will be integral to supporting equity in future infrastructure decisions.

## 1 Introduction

Infrastructure service outages are not uniformly distributed within communities but are often clustered disproportionately in areas with higher concentrations of socially vulnerable populations (e.g., low income and minority) with limited access to alternative sources, resulting in heightened adverse impacts. This pattern has been documented after Winter Storm Yuri (2021) for low‐income and minority groups (i.e., Black or African American and Hispanic) (Lee et al. 2022; Nejat et al. 2022), after Hurricane Irma (2017) for minorities, low‐income groups, and individuals dependent on medical devices (Chakalian et al. 2019; Mitsova et al. 2018; Mitsova et al. 2021), and after Hurricane Harvey (2017) for minorities, renters, low‐income households, and those with young children (Coleman et al. 2020). Neighborhoods with higher concentrations of socially vulnerable households are also often serviced by less resilient and less well‐maintained infrastructure that is more prone to outages (Highfield et al. 2014). The increased outage likelihood only serves to exacerbate disproportionate impacts generally felt by socially vulnerable populations (Hendricks and Van Zandt 2021; Van Zandt 2019; Williamson et al. 2023).

Disproportionate disaster impacts may diminish the positive effects of measures to enhance community disaster resilience. Thus, consideration of inequity stemming from disproportionate impacts is needed in community‐level disaster impact mitigation decision‐making. For this reason, equity is garnering more traction as a key element of community resilience (Cremen et al. 2022; Fletcher et al. 2022; Matin et al. 2018; National Academies of Sciences, Engineering, and Medicine 2022; Rendon et al. 2021). Metrics to quantitatively measure equity alongside other decision factors can support community resilience enhancement programs, such as the Building Resilient Infrastructure and Communities (BRIC) or Flood Mitigation Assistance (FEMA 2022). Although limited works incorporate disproportionate impacts into infrastructure decision‐making (AbdelMagid et al. 2023; Beck et al. 2025; Yang et al. 2023), even fewer explicitly quantify the state of inequity or changes due to decisions. An equity‐based infrastructure performance metric is therefore required to quantify and enable equity considerations alongside other system performance, cost, and temporal considerations commonly weighed in infrastructure decision‐making. In this work, we develop a novel equity‐based metric to evaluate the equity of infrastructure service performance between groups of concern for use in infrastructure decisions (e.g., retrofit, systems operation, restoration). The proposed metrics will better enable equity considerations to be assessed and integrated into the infrastructure decision paradigm in future applications.

Equity‐based metrics currently in use are often formulated to measure equality in resource distribution (i.e., distributional equity). These metrics strive to equally distribute a scarce resource across the entire population yet do little to identify or target groups who might face systemic inequities and disproportionate impacts (i.e., restorative equity). The metric developed herein supports decisions reducing systemic inequities and disproportionate impacts from the restorative equity paradigm while also supporting decisions from the distributional equity paradigm. Specifically, our metric targets distribution pole performance assessment to ultimately guide decisions to reduce service inequity for all household structures and associated households, yet the metric could be modified for applications to other infrastructure systems. The proposed restorative equity metric arises from Theil's *T* index (Theil 1967) and its decomposition features. Theil's *T* measures the inequality present in the distribution of a scarce resource, which accommodates distributional equity concerns. For the infrastructure application, the scarce resource can be defined via any infrastructure service performance measures (e.g., household service robustness and household service vulnerability). Most importantly, Theil *T*’s decomposition feature provides insight on the service performance differences between specific groups, enabling a restorative equity‐based decision assessment. Together these features make Theil's *T* a useful tool for decision makers to evaluate and adjust their decisions for both distributional and restorative equity‐based decisions.

The rest of the article is as follows. Equity and its five dimensions are defined with a brief literature review of the derived metrics currently in use. The methodology for the proposed restorative equity supporting metric, including its decomposition and relativization, is presented along with the definition of scarce resource in infrastructure decision‐making. The proposed metric's ability to capture degrees of inequity is assessed by application to a hypothetical electric distribution network (EDN) with varying nodal vulnerability scenarios. Then a discussion of the results and decision implications is presented, followed by the conclusions and future applications.

## 2 Equity Considerations in Infrastructure Decision‐Making

There is no standard definition of equity nor consensus on how equity should and can be applied broadly, let alone to infrastructure. Although equity has been operationalized within certain infrastructure sectors (e.g., water (Osman and Faust 2021) and transportation (Manaugh et al. 2015)), there can be sector‐specific attributes that are not applicable across infrastructures (e.g., right to clean water). Further, there can be contrasting perspectives on how equity should be defined. For example, one might assess equity at the input level (e.g., providing the same resources to all parties), whereas an alternative assesses equity at the output level (i.e., providing differential resources to account for initial differences, yielding the same outcomes). These alternative perspectives are encapsulated in McDermott et al.’s (2013) question for assessing equity: “what counts as equity?” (Kalra et al. 2022; McDermott et al. 2013). As a consequence, we offer the following discussion addressing the multiple dimensions and approaches for operationalizing equity with respect to infrastructure and clarify, which dimension this work strives to support.

### 2.1 Equity Dimensions

**FIGURE 1:**
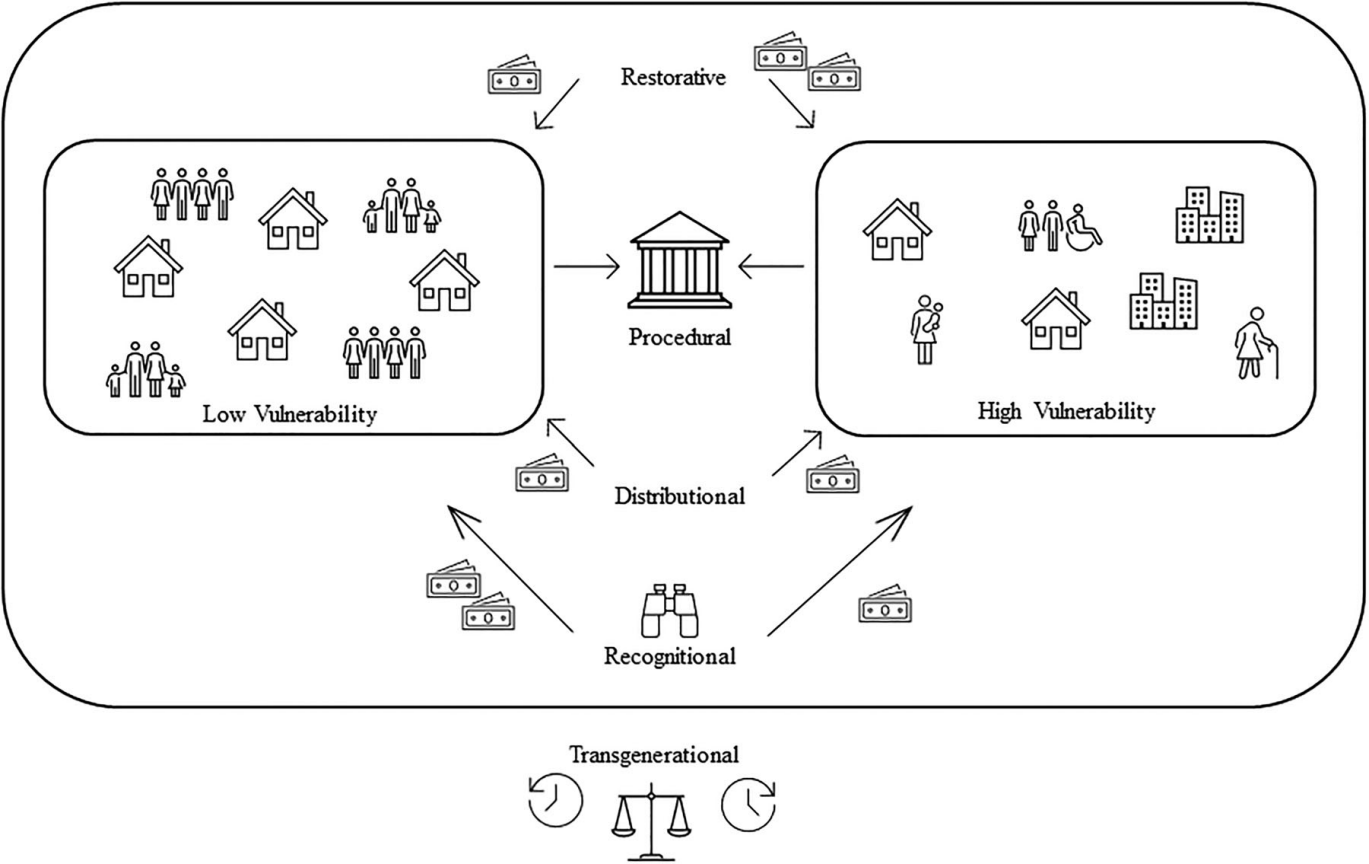
Conceptual diagram of equity.

Although there is no consensus on a singular definition of equity, more consensus is found for the dimensions of equity. There are five primary dimensions found across justice and equity literature: recognitional, distributional, restorative, procedural, and transgenerational. These dimensions are defined in Table 1, and Figure 1 offers conceptual illustrations. These dimensions originate from the climate, environmental, and energy justice fields and, as will be discussed, are relevant to the infrastructure decision process. Although some have divided equity into horizontal and vertical dimensions (Karakoc et al. 2020; Osman and Faust 2021), capturing distributional and restorative dimensions, the following provides a richer, multi‐dimensional characterization collectively emphasized by the literature to address equity.

**TABLE 1:** Equity dimension definitions.

Equity dimension	Definition	References
Recognitional	Equal awareness and identification of impacts or hardships across and among different groups	Bozeman et al. (2022), Lin et al. (2022), Meerow et al. (2019), Romero‐Lankao and Nobler (2021)
Distributional	Fair distribution of resources or service across all community groups.^a^ Often termed *equality*	Bozeman et al. (2022), Farley et al. (2021), Lin et al. (2022), Meerow et al. (2019), Park (2014), Romero‐Lankao and Nobler (2021), Fortman et al. (2022)
Restorative^b^	Prioritization of those who have been historically marginalized (and often still face marginalization) to rectify past and ongoing inequities^c^	Farley et al. (2021), Lin et al. (2022), Park (2014), Romero‐Lankao and Nobler (2021), Fortman et al. (2022)
Procedural	Inclusion and equal consideration of all equity perspectives in institutional decision‐making processes. Achieved through modified policies and rules that govern processes	Bozeman et al. (2022), Farley et al. (2021), Lin et al. (2022), Meerow et al. (2019), Park (2014), Romero‐Lankao and Nobler (2021), Fortman et al. (2022)
Transgenerational^d^	Fair distribution of burdens across generations. No unfair burden is placed on future generations or specific groups within a future generation	Park (2014), Romero‐Lankao and Nobler (2021), Fortman et al. (2022)^e^

^a^ Although some cite only equal distribution of resources, others (Farley et al. 2021; Y. Lin et al. 2022; Meerow et al. 2019; Park 2014; Romero‐Lankao and Nobler 2021) include burdens or benefits that can blend into restorative equity because those unfairly burdened tend to also be historically marginalized. In this study, distributional equity is viewed strictly as resource or service performance, whereas restorative equity addresses the socially vulnerable and the historically marginalized.

^b^ This dimension is sometimes termed structural.

^c^ This dimension of equity has been cited in contrast to equality in works such as Kim and Sutley (2021).

^d^ This dimension is sometimes termed cosmopolitan, which emphasizes the integration of energy life cycle across time coinciding with time transgenerational issues.

^e^ Fortman et al. (2022) does not explicitly name this equity dimension, but as part of their distributional definition, unfair burdens on future generations are included.

### 2.2 Current State of the Implementation of Equity in Infrastructure Decision‐Making

Individual equity dimensions have been uniquely operationalized within infrastructure decision‐making. The current implementation state of each equity dimension in infrastructure decision‐making is summarized below:

***Recognitional***: When adopted for infrastructure applications, recognitional approaches switch from a purely technical viewpoint to one that explicitly documents the disproportionate impacts of outages. This approach identifies areas of impact and quantifies the disproportionate impacts across groups. This identification process explicitly enables successive criticality assessment and prioritization that has the potential to address equity. Multiple studies have, for example, developed approaches for identifying differential impacts and their disproportionate consequences across socially vulnerable populations related to power outages (Batouli and Joshi 2022; Best et al. 2023; Coleman et al. 2023; Jiang et al. 2023). Additionally, impact quantification at infrastructure level has been supported by the outage social impact criticality analysis (Beck et al. 2025; Beck and Cha 2022) and other impact quantification methods at the household level (Clark et al. 2023). The combination of identification and quantification of inequities due to infrastructure failures enables recognition and an opportunity to mitigate future inequalities.

***Distributional***: The distributional paradigm holds that prioritization should be nondiscriminatory such that all should receive equal service performance. The equality of service performance has traditionally governed infrastructure decisions beyond the prioritization of critical facilities (e.g., hospitals and emergency management). With widespread implementation in infrastructure decisions, many metrics exist in support of distributional equity and can be broadly classified into three categories: (i) threshold‐based, (ii) gap‐based, or (iii) equality deviation–based.

*Threshold‐based metrics* benchmark system performance relative to a threshold level of service performance or service failure rate “equally” across a community. Therefore, operators justify striving for a threshold service level to equally improve service performance to an achievable level. In practice, some thresholds can be set where a system is required to meet minimum service levels across the system to a certain degree (i.e., 90%). This can allow system anomalies; however, some community parts may inadvertently be systemically within the service areas, not satisfying the threshold.

*Gap‐based metrics* capture the difference in service performance levels across a community. The goal is to reduce the gaps and equally improve aggregate performance. Gaps can be defined in any manner to support decision makers’ priorities (e.g., min–max), and these metrics can be tailored to highlight differences between vulnerable populations or neighborhoods. For example, France‐Mensah et al. (2019) and Kothari and O'Brien (2022) used absolute differences (i.e., gaps) in average pavement condition scores, across years, between disadvantaged and non‐disadvantaged groups.

*Equality deviation–based* measures capture the inequality in resource distribution across a community and how it deviates from a perfectly equal distribution. The Gini index (Gini 1921) and the Theil indices (e.g., Theil's *T* and Theil's *L*) (Theil 1967) are classic examples. The Gini produces a bounded value varying between 0 and 1, with 0 representing a perfectly equal distribution and 1 representing the corollary. Theil indices are unbounded and range from 0 to a maximum value of ln(N), where N is the number of observations. The Theil indices supporting distributional equity differ from the later presented decomposition terms (i.e., between‐zone inequality BZI] and within‐zone inequality [WZI]) that support restorative equity, which will be addressed more fully in subsequent sections. Equality deviation–based metrics have been implemented in infrastructure decision‐making with the goal of minimizing Gini or Theil's *T* to achieve the most equal infrastructure service performance in multiple infrastructure applications (Cullis and Van Koppen 2009; Dhakal and Zhang 2022; Gunasekara et al. 2014; Hu et al. 2016; Kothari and O'Brien 2022; Seyedashraf et al. 2022; Xu et al. 2019).

***Restorative***: The restorative paradigm for infrastructure dictates the prioritization of marginalized and vulnerable population subsets, which have been and are disproportionately serviced and impacted by infrastructure outages. This prioritization works to reduce future outage impact inequities and correct historic injustice perpetuated by inequitable, often systemic infrastructure disinvestment. The restorative equity paradigm to date has not been well supported by metrics and other decision support tools at resolutions required for infrastructure component‐level improvements. The previous metrics described for distributional equity are not well suited as they work to aggregately strive for equal distribution and do not support the prioritization of specific groups. Tools stemming out of the Justice40 initiative, such as CJEST, do support restorative equity prioritization but at a coarser level (i.e., census tract) for general identification of areas for prioritization (White House n.d.). The majority of these tools are not conducive for infrastructure decisions. Calls have been made for further development of the restorative equity paradigm (Barlow et al. 2022; Kalra et al. 2022; Tarekegne et al. 2021).

***Procedural***: The procedural paradigm dictates the formation and involvement of broader stakeholder groups in infrastructure decision‐making to promote more equitable outcomes. The process should be structured in a manner that permits all parties’ perspectives, including their risk aversions or neutrality, to be equally incorporated and critically assesses factors shaping decisions. This larger and more diverse group can introduce an increased number of contrasting perspectives and priorities that will often uncover or “recognize” heretofore unrecognized inequalities generated by seemingly neutral, formal, and allegedly objective procedures and processes. New decision support tools are therefore necessitated to support multiple stakeholders’ perspectives and facilitate dialogue, cooperation, and analyses of procedural outcomes.

***Transgenerational***: The transgenerational paradigm dictates that the traditional short‐term cost–benefit timeline should be extended to accommodate future‐looking and climate‐change cognizant perspectives. Infrastructure decisions should consider long‐term consequences beyond current generations, striving to reduce any burden unduly placed on future generations. Communities thus must achieve buy‐in relative to extended benefit periods more than traditionally expected. Thus, metrics and considerations should be modified and developed to better reflect time‐conscious transgenerational aspects.

This review identifies the many and diverse dimensions potentially involved in equity‐based decisions. The implementation of all dimensions in infrastructure decision‐making is a monumental interdisciplinary task. Of the dimensions, transgenerational is by far the most complex and difficult to operationalize and, as such, has few support tools currently. Distributional, on the other hand, is the most widely supported dimension with a great variety of possible tools and metrics. Recognitional is supported by a more limited number of examples. Even after differential impacts are recognized, having metrics to guide decision‐making is critical, and many distributional approaches and metrics would not be precluded from supporting the recognitional dimension. Similarly, procedural inequality could benefit from distributional or restorative metrics and approaches for deciding which actions might redress the issues. With both *recognitional* and *procedural* approaches, there is an implicit understanding that inequalities are being generated due to factors across specific categories or groups. Hence, we ultimately are concerned with *restorative* inequities across some dimensional attributes that must be addressed to restore equity. As such, our primary focus and effort of development pertains to the restorative dimension given its potential relevance for recognitional and procedural dimensions, its relevance for infrastructure decisions, and its scarcity of decision support tools to date. At the same time, distributional (i.e., equality) concerns are widely germane in practice and considered alongside restorative concerns.

## 3 An Equity Metric for Infrastructure Performance Assessment

This work derives new metrics capable of supporting the restorative equity paradigm by building on the Theil indices that assess the distributional equity. To support restorative equity‐based decisions, the strength of Theil indices is the ability to decompose these metrics and thereby assess increases or decreases in inequality across specific community attributes in response to mitigation or recovery decisions. The following subsections present the metric's derivations and specifications for restorative infrastructure decision‐making contexts.

### 3.1 Theil's Equity Metrics

Theil's indices are entropy‐based measures quantifying a scarce resource's dispersion from a perfectly equal state (Theil 1967). Originating in economics and derived from information theory, Theil offered two indices to capture household income inequality across various subgroups in a population: Theil's *T*, which evaluates inequality of a scarce resource weighted by the proportion of each group's resource share, and Theil's *L*, which evaluates inequality in the scarce resource weighted by the proportion of each group's population size. Although initially intended for income, both can be utilized to measure the inequality of any scarce resource. In this study, infrastructure equity metrics are derived where the scarce resource is measured by bounded measures, for example, service quality, rather than unbounded measures (e.g., monetary value). When weighting by the proportion of each group's population size, smaller population sizes are underweighted. These smaller populations also often intersect with populations of vulnerability, meaning these populations could be underprioritized. Hence, our focus here is on Theil's *T*.

Like the Gini index, Theil's *T* assesses inequality (i.e., distributional inequity); however, the latter was developed to capture that inequality relative to groups or zones within a population and is more aligned with the restorative equity paradigm. Theil's *T*, $T_{T}$, is defined by the following equation (Liao 2016; Peacock et al. 1988):

(1) $$T_{T}=\mathrm{BZI}+\mathrm{WZI}$$

(2) $$\mathrm{BZI}=\sum_{z=1}^{n}y_{z}\cdot \ln\frac{{\bar{x}}_{z}}{\bar{x}}$$

(3) $$\mathrm{WZI}=\sum_{z=1}^{n}y_{z}\cdot \left(\sum_{i=1}^{n_{z}}y_{iz}\cdot \ln\frac{x_{iz}}{{\bar{x}}_{z}}\right)$$

where BZI is the between‐zone inequality, which is the inequality attributed to differences between the groups considered, WZI is the within‐zone inequality, which is the inequality attributed to differences within all of the groups collectively, $\bar{x}$ is the overall mean scarce resource value across the whole community, $x_{iz}$ is the *i*th individual's or entity's scarce resource amount in the *z*th group, n is the number of groups considered, $n_{z}$ is the number of individuals in the *z*th group, $y_{z}$ is the *z*th group's (or zone's) proportion of scarce resource relative to the total amount of the resource defined as

(4) $$y_{z}=\frac{\sum_{i=1}^{n_{z}}x_{\mathrm{iz}}}{\sum_{i=1}^{n_{p}}x_{i}}\;$$

where *n*_*p*_ is the total number of individuals, and *x*_*i*_ is the individual's or entity's scarce resource amount. ${\bar{x}}_{z}$ is the *z*th group's mean scarce resource amount defined as

(5) $${\bar{x}}_{z}=\frac{\sum_{i=1}^{n_{z}}x_{\mathrm{iz}}}{n_{z}}\;$$

$y_{iz}$ is the *i*th individual's or entity's proportion of scarce resource relative to the *z*th group's total amount of scarce resource defined as

(6) $$y_{iz}=\frac{x_{iz}}{\sum_{i=1}^{n_{z}}x_{iz}}$$

$T_{T}$ gives the inequality present in the scarce resource's distribution and ranges from 0 to a maximum value of ln(N), with N being the number of individuals whose service performance is being considered. The larger the magnitude of Theil's *T*, the greater the inequality in the scarce resource's distribution (i.e., more distributional inequity). The $T_{T}$ value can be directly utilized to inform distributional equity‐based decisions across the whole community to reduce the overall amount of inequality. Zones are defined as any group of individuals or entities that share a significant attribute or set of attributes. The number of groups, k, can take on any value greater than 1. However, for implementation in the restorative context and for intuitive interpretation, k=2 is most appropriate. By only computing relative to two groups, targeted implementation to reduce the inequity is more straightforward. As more groups are considered, interaction effects could possibly complicate proper selection for mitigation efforts. The group formulation will thus be defined as a group comprised of individuals with characteristics of concern and its complement group (e.g., minority vs. non‐minority, low income vs. non‐low income), or even compound vulnerability (i.e., low income and minority status, low income and majority status, and high income and majority status). As such, BZI indicates the amount of total inequality due to scarce resource inequality between these two groups, whereas WZI indicates the amount of total inequality due to scarce resource inequality within the groups. A larger value in either term indicates more of the inequality is attributed to between‐ or within‐zone differences. In the context of restorative inequality, BZI becomes highly salient.

Because restorative equity dictates the prioritization of groups who have been systemically or historically marginalized and face disproportionate impacts relative to their non‐marginalized counterparts, a restorative equity‐based decision strives to reduce the amount of inequality between two groups. Hence, the BZI component directly enables decision makers to assess inequality between groups to inform decisions under the restorative equity paradigm. A larger BZI component indicates there is a greater degree of inequality between groups. Additionally, changes to BZI after a decision can gauge success in reducing inequality. The $T_{T}$ within each group could also be quantified to understand distributional differences within groups, which is mentioned herein for completeness but not addressed within this study for brevity. $T_{T}$ and its decomposed metric BZI enable both distributional and restorative equity‐based decision‐making, respectively, depending upon how the groups are defined, allowing for both equity dimensions to be considered.

A potential shortfall of $T_{T}$ and its decomposed metrics is its upper bound being dependent on the number (N) of individuals or entities being considered. This characteristic has made $T_{T}$ less popular than Gini. To overcome this commonly cited shortfall, the maximum, ln (N), value can be employed to standardize $T_{T}$ between 0 and 1 to facilitate comparisons across implementations. The relative $T_{T}$, $T_{T}^{\mathrm{rel}}$, is defined as follows:

(7) $$T_{T}^{rel}=\frac{T_{T}}{ln(N)}$$

A $T_{T}^{\mathrm{rel}}$ of 1 represents complete inequality, whereas 0 represents equality. Given the additive property of the decomposition elements of $T_{T}$, relative component terms are defined as follows:

(8) $$\mathrm{BZ}I^{\mathrm{rel}}=\frac{\mathrm{BZI}}{T_{T}}$$

(9) $$\mathrm{WZ}I^{\mathrm{rel}}=\frac{\mathrm{WZI}}{T_{T}}$$

The relative decomposition metrics represent the fraction of inequality attributed to differences between or within groups, respectively. A BZI or WZI value closer to 1 indicates that total inequality is attributable to differences between or within groups, respectively. These relative metrics are more readily interpretable, can more easily inform restorative equity‐based decision‐making, and will be employed throughout our discussions. It should be emphasized that the relative decomposition metrics are inherently related to the magnitude of $T_{T}$. Their evaluation will require a tandem evaluation of $T_{T}$ to determine if the restorative inequity detected is of great enough magnitude relative to overall inequality (i.e., distributional inequity). This necessity will be emphasized in the application section but is included here for completeness.

Other beneficial features render Theil's *T* an advantageous metric to assess equity. First, Theil's *T* is scale invariant, meaning that the scarce resource's input units do not alter inequality assessments if units are held constant across all inputs. Second, Theil's *T* upholds Dalton's principle of transfers because inequality is reduced when scarce resources are transferred from individuals with greater resources to those with lower resource amounts (Dalton 1920). The principle of transfers is relevant in infrastructure applications due to budget scarcity and variations in infrastructure quality across a system. The scarce resource for infrastructure applications is influenced by many factors, as described in the next section, and as such the transfer phenomenon is not as readily apparent compared to income‐based applications. With budget scarcity dictating infrastructure state and initial state, along with improved state, the transfer can be logically outlined with assessing service performance gains. Improvements in one area may preclude performance gains elsewhere, producing transfer‐like dynamics that can be traced through relative performance gains. Finally, Theil's *T* exhibits transfer sensitivity at the lower end of the scarce resource scale, where such transfers have greater marginal utility (Shorrocks 1980; Shorrocks and Foster 1987). This means that Theil's *T* displays greater sensitivity to resource transfers between individuals at the lower end of the resource spectrum when compared to those at the upper end. Together, these final two features, particularly given the measure's ability to capture the relative importance of between‐group inequality's contribution to total inequality, better ensure the overall ability of Theil's *T* to capture distributional inequality and to monitor changes in response to policy decisions to address restorative inequity.

### 3.2 Scarce Resource for Infrastructure Applications

The critical step to transform Theil's *T* and its derived metrics from economics to infrastructure applications is defining the scarce resource. The designation of scarce resources should meet three primary criteria: (i) The scarce resource should be indicative of service performance (i.e., service quality); (ii) the scarce resource should be assessed at sufficiently high resolution, allowing decomposition between entities, such as households, that can be categorized into socio‐economic or demographic vulnerable groups of concern; and (iii) the scarce resource should capture compounding systemic vulnerability. With respect to the final criterion, service performance inequity is not due to one faulty component but is often attributable to compounding systemic and historic infrastructure disinvestment. Although measures such as the age of the closest infrastructure component are indicative of individual component quality, they neglect the compounding system vulnerability that can alter the realized service performance quality. As such, a system‐based formulation of service performance quality is preferred to account for compounding vulnerabilities.

Considering the requirements, two system‐based service performance measures are used in this study: (i) robustness (i.e., probability of household service performance) and (ii) vulnerability (i.e., probability of household service outage). These two quantities represent a complementary set. Robustness represents a positive ordinated scarce resource, whereas vulnerability is a negative ordinated scarce resource. A positive ordinated scarce resource is one in which higher levels are desirable, whereas the corollary is true of a negative ordinated one, and in the infrastructure case, both formulations are implemented to capture performance differences. Additionally, the mitigation of negative ordinated vulnerability is often a primary concern for decision makers and justifies the initial investigation into this less traditionally ordinated scarce resource. Robustness, $P_{\mathrm{service}}$, is formulated through series system reliability, where all critical components’ functioning is required for service performance at an entity's structure as follows:

(10) $$P_{\mathrm{service}}=\prod_{i=1}^{m}P_{s}^{i}$$

where $p_{s}^{i}$ s are the individual components’ probabilities of survival, respectively, and m is the number of components in an individual structure's shortest path for critical service performance. The collective evaluation of all components functioning or failing through series system formulation constitutes a simple accountment of compounding system vulnerability, which could be expanded in the future. Each component's survival probability could be determined empirically, through testing, or through assumptions. This formulation does neglect network redundancies, and a modified parallel system could be implemented to account for all redundancies. However, the computational burden to outline all possibilities is infeasible with current computational capabilities and frameworks. Currently, outages have been found to be of longer duration for those of greater socioeconomic vulnerability (Best et al. 2023). From this and reported experience, network redundancies are touted to be clustered outside of areas with the greatest vulnerability, leading to longer outage durations. Therefore, consideration of redundancies would only further exacerbate service performance disparities. Therefore, the series system serves as a lower bound estimate for robustness.

Vulnerability, $P_{\mathrm{outage}}$, is formulated as follows based on its definition as the complement to robustness:

(11) $$P_{\mathrm{outage}}=1-P_{\mathrm{service}}$$

These are just two examples of scarce resource formulations for infrastructure considered in this study. Any other scarce resource could be adopted if justification and rationale align with the decision maker's interest. In the future, this formulation could be expanded to consider additional components (i.e., lines, conductors) in addition to poles themselves or to account for correlation between adjacent pole failures. The scarce resources ($P_{\mathrm{service}}$, $P_{\mathrm{outage}}$) are calculated for final service units or customers, generally businesses, housing units, or households residing in those units, to define $x_{iz}$, and the derived terms (i.e., the proportion of the scarce resource share of a group [$y_{z}$] and mean scarce resource of a group [${\bar{x}}_{z}$]) required for Theil's *T* can be calculated.

## 4 Equity Metric Application and Verification

### 4.1 Hypothetical Trial Area and Application Description

**FIGURE 2:**
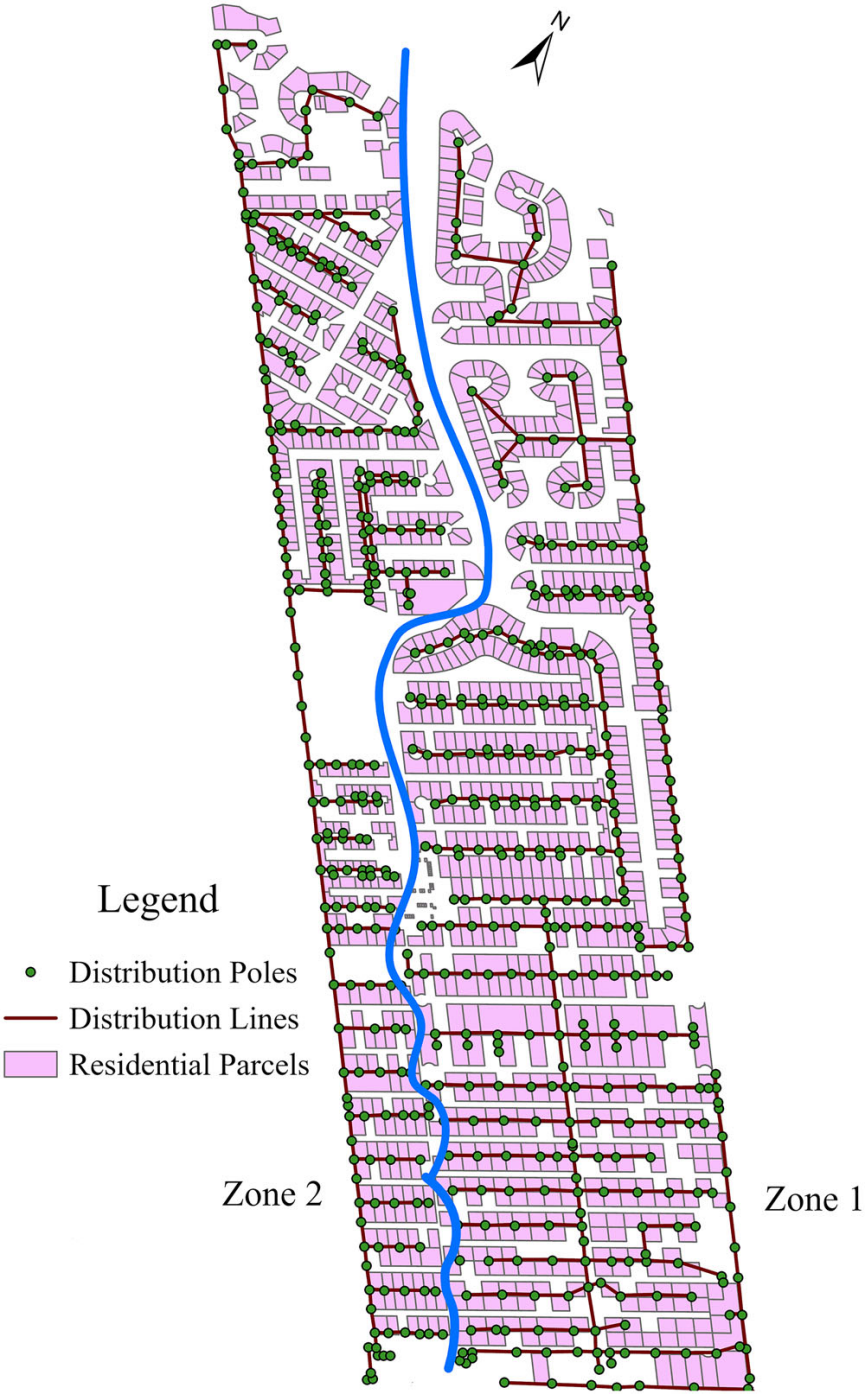
Study area schematic and zone division.

The equity metric is implemented for a hypothetical compact EDN shown in Figure 2 to assess the equity in service performance to households. This study area is derived from a subregion of Galveston, Texas's EDN from Darestani and Padgett (2022) and housing unit locations are derived from Galveston County's assessor's data (Galveston Central Appraisal District n.d.). This region is subjected to extreme wind events, such as hurricanes, and the EDN's performance is assessed relative to wind hazard. Although hurricane events are multi‐hazard with wind–surge–wave intensities, the singular hazard consideration is deemed proper for initial implementation and appropriate due to a lack of hurricane multi‐hazard intensity distribution characterization. This small test area is selected for initial application to understand the metrics’ innate behavior and variations in equity detection performance.

In this study, inequity is assessed relative to two zones, Zones 1 and 2, which are defined on the basis of the EDN's topology. Zone 1 consists of approximately 70% of the distribution poles linked to approximately 65% of the housing units, with the main distribution line feeding into the bottommost‐right component. Zone 2 is a smaller zone consisting of approximately 30% of the distribution poles linked to approximately 35% of the housing units, with the connection to the main distribution line feeding the sub‐net from the bottom left. Although inequity in service performance could be assessed between demographic groups of concern (e.g., low income vs. non‐low income and minority areas vs. non‐minority areas), as is often the case, it is not the sociodemographic or economic characteristics themselves that are driving inequities, but social and economic processes shaping the nature, state, or quality of infrastructure that is the real issue. In the future, demographic divisions would ideally be based on demographic divisions given available characterizations rather than geographic groupings; the application herein reinforces the generalizability of the method for any division of concern.

For this study, age is taken as the singular indicator of pole quality, with older poles having worse performance. Age is an appropriate parameter to employ in our example, because neglect in infrastructure maintenance and investment (i.e., disinvestment) can be introduced by older poles with lower quality. Therefore, patterns of pole age are manipulated to simulate varying service performance/failure probabilities. The performance of individual distribution poles is characterized by employing Darestani and Shafieezadeh's (2019) fragility model, which includes an age parameter for determining pole performance. Other pole and input characteristics will be held constant. Specifically, all poles are assumed to be Class 5 with a common height of 25 ft (7.62 m), the wind direction angle is assumed to be 90°, which represents the worst‐case scenario, and the conductor area is assumed to be 2 m^2^. The fragility model produces no performance variability between ages 0 and 25 years, representing the ideal baseline performance levels for this study; however, as poles age, the failure probabilities increase, holding other factors constant.

For purposes of this study, several scenarios with similar and different age compositions of poles, generating heterogeneous performance (i.e., failure probabilities), are employed to assess our Theil's *T*‐based metric's ability to capture inequities between the zones.

### 4.2 Household Scarce Resource Calculations

Both scarce resource formulations, robustness and vulnerability, are dictated by the networked connection between the origin component (i.e., substation) and the base component of service (i.e., distribution pole). For this study, we adopt Beck and Cha's (2022) characterization of a base service component (or node) as the terminal component most proximate to a structure (i.e., housing unit) requiring the service it provides so that the structures’ occupying social unit (i.e., household) can undertake its normal functions. The identification of base components is implemented using ArcGIS's nearest neighbor function. With the base component for each household and the origin component of service, the critical service path to compute the probability of services is defined for each housing unit by the shortest path (path with the least number of poles) between the base component and the origin component of service. Only distribution poles are considered critical components in the service performance. Although other components such as conductors and lines could potentially be considered, extant fragilities for these components are limited, and their inclusion is not critical for an assessment of testing the utility of our metric. The origin component in this application is set as the component at either corner of the network, representing the connection to mainline distribution lines. When available, a higher level component, such as a substation, could serve as the ideal origin component so that the system analysis would more fully account for all components critical for service.

The individual component survival likelihood is found by jointly accounting for component failure uncertainty and hazard intensity uncertainty via the total probability theorem. However, component survival likelihood could be specified by decision makers based on known or assumed component vulnerabilities. The formulation adopted in this application is displayed as follows:

(12) $$P_{s}=1-\int_{R}P\left(outage|v\right).f\left(v\right)dv$$

where P(outage|v) is the distribution pole fragility and f(v) is the extreme hazard intensity probability density function for hazard intensity v. In this application, the hazard is wind, and Yeo et al.’s (2014) estimated reverse Weibull extreme wind characterization for Galveston (TX, USA) is implemented. With the characterization and survival likelihood of each individual component in addition to their respective critical service paths, each household structure's scarce resources can be calculated via Equations (10) and (11), respectively.

### 4.3 Metric Behavior and Variation in Equity Detection Performance

The relative version of Theil's *T* is employed to assess the metric's abilities to detect inequality, its sensitivity given various degrees of inequality, and its utility given both robust and vulnerability scarce resource definitions. To facilitate these assessments, two scenarios in which alternative age of distribution poles within and between the two zones are employed. These are presented in Table 2. Under the first scenario, a uniform distribution of poles by age categories between the two zones is assumed and implemented. The second scenario will offer two sub‐scenarios with two different age distributions between the two zones. In the first sub‐scenario, 2a, Zone 1 has baseline, 0‐ to 25‐year poles, whereas Zone 2 has progressively older sets of poles. In the second sub‐scenario, 2b, Zone 2's poles are all baseline ages, and Zone 1 has the progressively older pole sets. Overall, these results affirm Theil's *T*‐based metrics provide meaningful detection of inequity, enabling both restorative and distributional equity‐based decision‐making. Key findings, which will be discussed more in depth in subsequent sections, are as follows: (i) Metrics aptly detect introduced inequity; (ii) $\mathrm{BZ}I^{\mathrm{rel}}$ is sensitive to size and measures higher for a smaller vulnerable minority, which supports restorative equity concerns; (iii) non‐linearity exists in metrics across introduced inequity; and (iv) distributional equity measure ($T_{T}^{\mathrm{rel}}$) is sensitive to scarce resources’ definition (i.e., ordination).

**TABLE 2:** Scenario age assignments.

Scenarios	Zones	Pole ages (years)				
1. Uniform	Zone 1	0–25	40	60	80	100
	Zone 2	0–25	40	60	80	100
2a. Varying	Zone 1	0–25	0–25	0–25	0–25	
	Zone 2	40	60	80	100	
2b. Varying	Zone 1	40	60	80	100	
	Zone 2	0–25	0–25	0–25	0–25	

#### 4.3.1 Inequity Detection Capability of the Metrics

The metrics’ capability in detecting inequity can be demonstrated by comparing their values between the uniform and varying pole age scenarios. When inequity is introduced by varying age scenarios, the metrics should respond by reflecting higher values of inequality. Additionally, as noted above, the relative sizes of the zones will also be important to consider in that the number of parcels in Zone 1 is significantly larger than that of Zone 2. The metric's values are displayed in Table 3 for the uniform age scenario and Table 4 for the varying age scenarios for both scarce resource considerations: vulnerability and robustness.

**TABLE 3:** Theil's *T*‐based inequity metrics for uniform pole age assignment.

Age (years) of poles in Zone 1	0–25	40	60	80	100
Age (years) of poles in Zone 2	0–25	40	60	80	100
Zone age difference (years)	0	0	0	0	0
Scarce resource—Vulnerability					
$T_{T}^{\mathrm{rel}}$	0.0170	0.0167	0.0127	0.0014	0.0000
$\mathrm{BZ}I^{\mathrm{rel}}$	0.0074	0.0073	0.0053	0.0011	0.0003
$\mathrm{WZ}I^{\mathrm{rel}}$	0.9926	0.9927	0.9947	0.9989	0.9997
Scarce resource—Robustness					
$T_{T}^{\mathrm{rel}}$	0.0000	0.0000	0.0026	0.1301	0.4204
$\mathrm{BZ}I^{\mathrm{rel}}$	0.0080	0.0079	0.0059	0.0018	0.0019
$\mathrm{WZ}I^{\mathrm{rel}}$	0.9920	0.9921	0.9941	0.9982	0.9981

**TABLE 4:** Theil's *T*‐based inequity metrics for varying pole age assignment.

	Scenario 2a				Scenario 2b			
Age of Zone 1 (years)	0–25	0–25	0–25	0–25	100	80	60	40
Age of Zone 2 (years)	40	60	80	100	0–25	0–25	0–25	0–25
Zone age difference (years)	15	35	55	75	75	55	35	15
Scarce resource—Vulnerability								
$T_{T}^{\mathrm{rel}}$	0.0316	0.1218	0.1346	0.1343	0.0556	0.0567	0.0622	0.0310
$\mathrm{BZ}I^{\mathrm{rel}}$	0.4843	0.8971	0.9858	0.9970	0.9974	0.9737	0.7915	0.4532
$\mathrm{WZ}I^{\mathrm{rel}}$	0.5157	0.1029	0.0142	0.0030	0.0026	0.0263	0.2084	0.5468
Scarce resource—Robustness								
$T_{T}^{\mathrm{rel}}$	0.0000	0.0025	0.0444	0.0588	0.1473	0.1032	0.0042	0.0000
$\mathrm{BZ}I^{\mathrm{rel}}$	0.4826	0.7686	0.8800	0.9894	0.9739	0.8423	0.6193	0.3970
$\mathrm{WZ}I^{\mathrm{rel}}$	0.5174	0.2314	0.1200	0.0106	0.0261	0.1577	0.3806	0.6030

**FIGURE 3:**
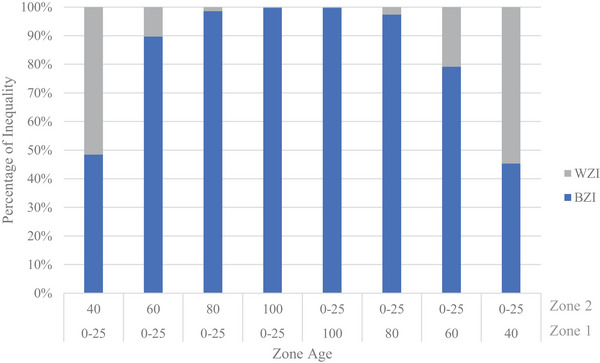
Theil's *T* inequality decomposition into relative WZI and BZI for varying zone age assignments for vulnerability scarce resources. BZI, between‐zone inequality; WZI, within‐zone inequality.

**FIGURE 4:**
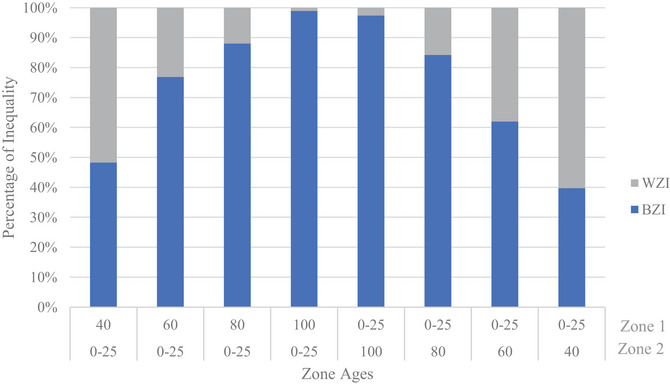
Theil's *T* inequality decomposition into relative WZI and BZI for varying zone age assignments for robustness scarce resources. BZI, between‐zone inequality; WZI, within‐zone inequality.

In most uniform age scenarios, the $T_{T}^{\mathrm{rel}}$ values are negligible and depict the introduced equality state as expected. When inequity is introduced by varying pole ages and compared with the uniform age scenarios, the $T_{T}^{\mathrm{rel}}$ and $\mathrm{BZ}I^{\mathrm{rel}}$ are increased significantly. This is indicative of the metrics detecting the inequity introduced in the varying age scenarios. In the 80‐ and 100‐year uniform cases for robustness, a large $T_{T}^{\mathrm{rel}}$ is present. We refer the reader to our discussion in Section 4.3.3, which will explore this phenomenon attributed to scarce resource definition more fully. Further, the detection of inequity between zones is seen through the decomposition terms. The $\mathrm{BZ}I^{\mathrm{rel}}$ component significantly and quickly increases with the increasing age gap in the varying age scenarios compared to the uniform scenarios. This change in value is seen in the decomposition values reported in Table 4 and displayed in the decomposition plots shown in Figures 3 and 4 for vulnerability and robustness, respectively. With an increase in age gap, the increased $\mathrm{BZ}I^{\mathrm{rel}}$ demonstrates the metric's ability to detect changes in inequity between groups and can inform restorative equity‐based decisions that work to reduce this difference between groups. Moreover, the larger $\mathrm{BZ}I^{\mathrm{rel}}$ values indicate that between‐zone differences contribute the most to $T_{T}^{\mathrm{rel}}$ compared to within‐zone contributions.

#### 4.3.2 Metric Sensitivity to Introduced Inequity

**FIGURE 5:**
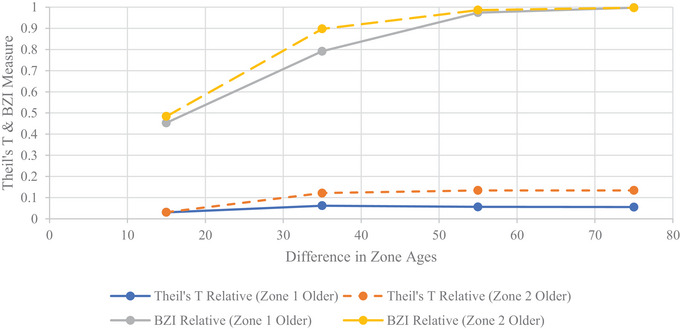
Theil's *T* and BZI relative values across differences in zone age assignments for vulnerability scarce resource. BZI, between‐zone inequality.

**FIGURE 6:**
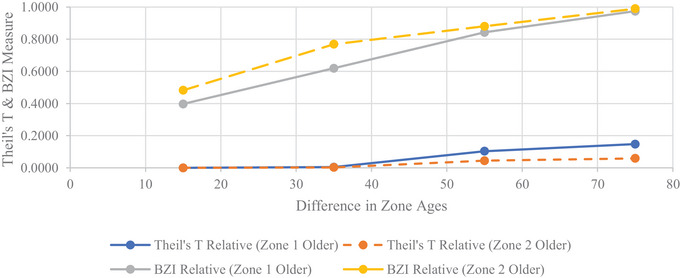
Theil's *T* and $\mathrm{BZ}I^{\mathrm{rel}}$ values across differences in zone age assignments for robustness scarce resources. BZI, between‐zone inequality.

More inequity is indicated by the higher $\mathrm{BZ}I^{\mathrm{rel}}$ when a fewer number of individuals receive lower service levels compared to their counterparts in a larger region with more individuals. Figures 5 and 6 display the plots of $T_{T}^{\mathrm{rel}}$ and $\mathrm{BZ}I^{\mathrm{rel}}$ as the zone age difference changes for vulnerability and robustness, respectively.

In these plots, it is shown that the $\mathrm{BZ}I^{\mathrm{rel}}$ values are greater when Zone 2 is older compared to when Zone 1 is older. Zone 1 constitutes approximately two‐thirds of the network region, whereas Zone 2 only occupies one‐third. When this slightly smaller region is serviced by older components and receives lower service levels, the between‐zone inequity decomposition is larger relative to the corollary. This characteristic is important for the investigation and implementation of restorative equity concerns, during which a vulnerable minority is often considered against a less‐vulnerable majority. This further affirms the metrics’ affinity to enable restorative equity considerations. Although visual inspection of these plots does not reveal a break in this trend, inspection of the tabulated values in Table 4 reveals an exception for 75‐year age difference scenarios for the vulnerability scarce resource, where $\mathrm{BZ}I^{\mathrm{rel}}$ of Zone 1 varied older is 0.0004 larger than Zone 2 varied older. Yet, in a practical sense, this difference is within range to be considered equal to Zone 2 varied older decomposition. The sensitivity to population size was not consistently observed for $T_{T}^{\mathrm{rel}}$.

Furthermore, nonlinearity is exhibited in inequity detected across the zone age difference. This is exhibited in Figures 5 and 6, as discontinuities are seen across the age differences. Even between scarce resource definitions, the rate of change in both $\mathrm{BZ}I^{\mathrm{rel}}$ and $T_{T}^{\mathrm{rel}}$ does not change at the same rate.

#### 4.3.3 Distributional Equity Metric Sensitivity to Scarce Resource

The inequality (i.e., distributional inequity) indicated by the $T_{T}^{\mathrm{rel}}$ exhibits particular sensitivity to the scarce resource definition between scenarios that is not evinced to the same degree by the decomposition term, $\mathrm{BZ}I^{\mathrm{rel}}$. Due to this increased sensitivity, scarce resource selection is critical, particularly if distributional equity is a primary concern. This sensitivity is seen with a larger $T_{T}^{\mathrm{rel}}$ when Zone 2 (smaller population) is varied older with vulnerability as the scarce resource and the corollary with a larger $T_{T}^{\mathrm{rel}}$ when Zone 1 (larger population) is varied older with robustness as the scarce resource. This is demonstrated in Figures 5 and 6, where we see a switch in which scenarios (i.e., Zone 1 older vs. Zone 2 older) $T_{T}^{\mathrm{rel}}$ have the greatest values. A switch also occurs between the rate of change in $T_{T}^{\mathrm{rel}}$ between the scarce resource definitions, where for vulnerability it initially changes before becoming constant, whereas corollary occurs for robustness. These switches are attributed to $T_{T}^{\mathrm{rel}}$’s sensitivity to lower‐end transfers.

**FIGURE 7:**
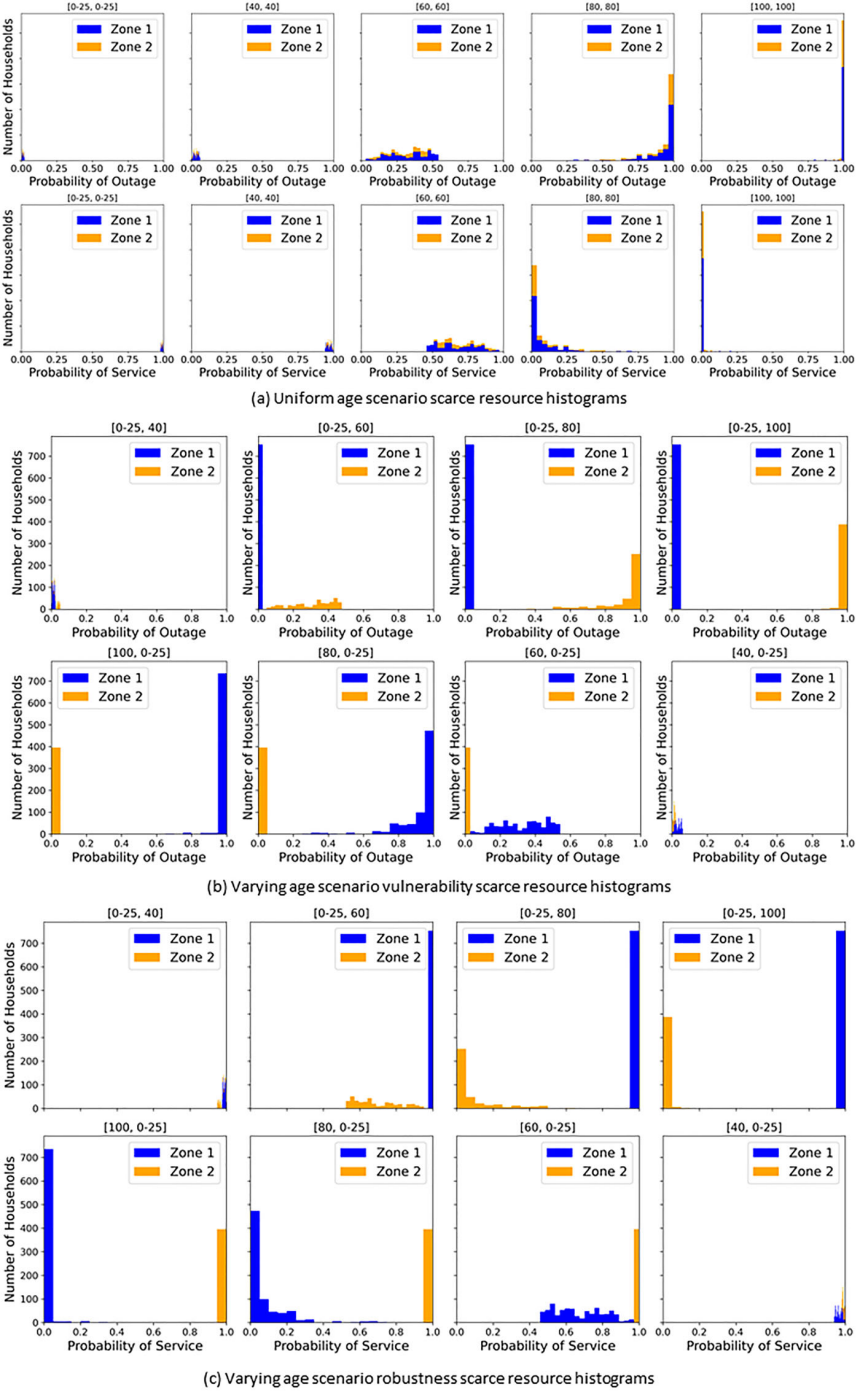
(a) Uniform age scenario, (b) varying age scenario vulnerability, and (c) varying age scenario robustness scarce resource histograms.

This sensitivity is confirmed by the varying scenario's scarce resource histograms for vulnerability in Figure 7b and robustness in Figure 7c. The largest $T_{T}^{\mathrm{rel}}$’s occurrence for each scarce resource coincides with the scenario with the greatest concentration of scarce resource around 0. When comparing the histograms with the opposite case (i.e., [0–25, 100] vs. [100, 0–25]), the transfer of resource distribution shifts toward 0 and the lower end of distribution scale, which Theil's *T* is known to register as more inequality, as discussed in Section 4.1. The clustering around 0 in opposite cases is due to the scarce resources being complements of each other and, as such, oppositely ordinated. Therefore, the scenario with the greatest concentration around zero occurs oppositely across the zone age variation due to the scarce resources’ intrinsic formulation. The distributional equity sensitivity therefore occurs due to scarce resources’ adopted ordination, and in that scenario, it will produce resource measures around 0.

Further, this sensitivity phenomenon also attributes for the extreme $T_{T}^{\mathrm{rel}}$ values seen in the robustness 80‐ and 100‐year uniform scenarios, where almost all the scarce resources are concentrated around 0. This concentration is evident in the histogram of scarce resource distributions shown in Figure 7a for both scarce resources, with the vulnerability scarce resource in the top row and the robustness scarce resource in bottom row. In these scenarios, the individual robustness rapidly and significantly declines for all poles. Yet, when these poles’ individual robustness are utilized collectively to characterize each household's robustness (i.e., probability of service) through a systems characterization, the households farther downstream have more rapid degradation of their robustness compared to households closer to the origin component. $\mathrm{BZ}I^{\mathrm{rel}}$ is falling with $\mathrm{WZ}I^{\mathrm{rel}}$ rising, indicating its inequality within the zone driving the increased $T_{T}^{\mathrm{rel}}$. With vulnerability as the scarce resource, these systematic inequality increases are not observed.

Due to sensitivity discussed above, it is apparent that the chosen scarce resource impacts how equity is assessed, particularly for distributional equity concerns. The two scarce resources evaluated in this study are simply two examples, one of positive ordination and one of negative ordination. Yet, decision makers could select alternative infrastructure scarce resources more pertinent to their decision problems at hand. It is therefore important for decision makers to critically evaluate what scarce resource is best suited for their implementation and ordination of that resource. For that end, we will briefly discuss the ramifications of each scarce resource in this study. However, this should by no means be taken as an exhaustive discussion nor investigation. Yet, this brief discussion can provide decision makers starting insight to consider as they select their scarce resource.

The adoption of robustness as the scarce resource due to its positive ordination exhibits increased logical coherence and rationality compared to the adoption of the negative ordinated vulnerability. A higher robustness value coincides with a more desirable condition, whereas a lower value coincides with a less desirable condition. Although it was initially surprising that a robustness approach was more sensitive to disparities, it perhaps should not have been because this matches the scarce resource paradigm exhibited by Theil's original scarce resource, income. The corollary is true for vulnerability, and a higher probability coincides with the less desirable condition. Although this scarce resource necessitates a counterintuitive paradigm, vulnerability can be taken as a proxy for outage burden (e.g., hardship). Especially when restorative equity concerns are in consideration, managing the hardship burden represented by the probability of outage can prove critical to decision makers. Therefore, some decision makers may find it more meaningful to evaluate infrastructure relative to this scarce resource with an increased difficulty associated with the counterintuitive oppositely ordinated paradigm.

The equity dimension of greatest importance to decision‐makers should also influence scarce resource selection. With $T_{T}^{rel}$ exhibiting this additional sensitivity, especially if decision makers are primarily concerned with distributional equity, more care should be placed in scarce resource selection considering their goal, either equal distribution of resource or burden. Although with the decomposition terms not exhibiting this increased sensitivity, when restorative equity is the primary paradigm of interest, scarce resource selection may not prove as critical without the exhibited flip, and both scarce resources could be readily utilized.

### 4.4 Considerations for Retrofit Application

The application and verification affirm the metrics’ ability to assess inequity and will facilitate equity assessments to guide infrastructure decisions. The presented metrics could be utilized to assess the inequity between groups of concern across a set of retrofit alternatives or implemented within optimization frameworks to minimize inequity of concern while devising optimal retrofitting plans. Specifically, decision makers could select just the $T_{T}^{\mathrm{rel}}$ if interested in the distributional equity paradigm, $\mathrm{BZ}I^{\mathrm{rel}}$ if interested in restorative equity paradigm, or both if interested in addressing both dimensions through their enacted decisions. The metrics of concern in each application should be computed for pre‐ and post‐retrofit for each proposed retrofit scenario to determine if and to what degree the equity is improved. When implemented in practice, these metrics should be implemented across an entire community and its whole state and not implemented within any piecemeal or superposition framework, as the additive nature of these metrics is far from accurate due to their innate structure and reliance on an entire population's scarce resource characterization. The metrics could be imbedded as performance‐based optimization objectives, where $\mathrm{BZ}I^{\mathrm{rel}}$ is minimized across multiple retrofit scenarios to reduce the overall restorative inequity. After initial verification of metrics’ ability to assess service performance inequities, future work will further explore implementation in retrofitting.

## 5 Conclusions and Future Work

Equity considerations are becoming of increasing importance in infrastructure decision‐making. Although there is no consensus on what constitutes equity, equity can be defined by five dimensions: recognitional, distributional, restorative, procedural, and transgenerational. The restorative dimension is of primary focus within this work and dictates the prioritization of marginalized and vulnerable population subsets that have been and are disproportionately serviced and impacted by infrastructure outages. To support restorative equity, a metric is developed to be integrated into infrastructure decision‐making alongside classic decision parameters of cost and system performance.

Theil's *T*‐based infrastructure metrics are devised relative to two scarce resources for infrastructure service performance quality represented through robustness and vulnerability. The derived metrics can support decisions based on both restorative equity and distributional equity decision paradigms. Distributional equity is supported by Theil's *T*, which measures the overall amount of inequality present in a scarce resource's spread. Restorative equity is supported by the decomposition term $\mathrm{BZ}I^{\mathrm{rel}}$, which indicates the inequality present between contrasting groups. The metric is implemented into a compact EDN network relative to a potential wind hazard where the inequity is assessed between two network zones with varying degrees of introduced inequity by varying pole age assignment. Overall, the results from implementation confirm that the derived metrics accommodate both restorative and distributional concerns and the metrics are sensitive to the degree of inequity introduced. However, much attention should be paid toward the scarce resource formulation as application for distributional dimension could be impacted.

The equity metrics should be further investigated and more completely integrated into a full infrastructure decision problem to understand how equity‐based considerations could alter retrofit selections among other economic, performance, and temporal considerations. Additionally, the metrics should be applied across networks of varying scales and characteristics and with different group designations to better understand their susceptibility and features. This will be better supported as high‐resolution data for both infrastructure and community demographics becomes more readily available or simulated. Lastly, alternate decompositions should be explored, both across demographics and further divisions across the network. Due to series system's scarce resource formulation, variation occurs due to network distance from the origin component. This could skew equity assessment and should be assessed within demographic considerations. Even with the current uncertainties in the metrics characterization and implementation, this work and its metric serve as one of the first decision supports for restorative equity considerations and further support equity's inclusion in infrastructure decision‐making. Further developments, particularly for the procedural and transgenerational dimensions, are relegated to future work when robust interdisciplinary collaboration and implementation can be pursued.

## Conflicts of Interest

The authors declare no conflicts of interest.
